# The ability to withstand short-term heat shocks and long-term elevated ambient temperatures suggests different sensitivity to future climatic changes for two sympatric Mediterranean land snail species, *Theba pisana* and *Xeropicta derbentina* (Helicoidea)

**DOI:** 10.1186/s12862-026-02527-7

**Published:** 2026-05-08

**Authors:** Heinz-R. Köhler, Yvan Capowiez, Line Capowiez, Rita Triebskorn

**Affiliations:** 1 Animal Physiological Ecology, Institute for Evolution and Ecology, University of Tübingen, Auf der Morgenstelle 5, D-72076 Tübingen, Germany; 2 INRAE, UMR Eco&Sol, Campus La Gaillarde, Bâtiment 12, 2 place Viala, Cédex 2, Montpellier, 34060 France; 3 INRAE Avignon University, UMR SQPOV, 228 avenue de l’aérodrome, Cedex 9, Avignon , 84914 France; 4 Steinbeis-Transfer Centre for Ecotoxicology and Ecophysiology, Blumenstraße 13, Rottenburg, D-72108 Germany

**Keywords:** Gastropoda, Global warming, Heat hardening, Heat tolerance, Open-top chambers, Shell

## Abstract

In the 1940s, *Xeropicta derbentina* was introduced to the Provence region in south-eastern France. Since then, it has coexisted sympatrically with the native species *Theba pisana*. *X. derbentina*‘s successful establishment in Provence is commonly attributed to the hot and dry climate there. In the context of continually rising temperatures and increasing aridity resulting from global change, we conducted open-top chamber (OTC) experiments with both species to simulate extreme heat conditions to be expected in the context of climate change. After exposure in the OTCs, the temperatures on the shell surface did not differ between the two species. Nevertheless, differences in the survival of the species following heat shocks, which were induced by transferring the snails to the hot soil, were striking. An initial heat shock at the start of the OTC exposure was survived significantly better by *T. pisana* than by *X. derbentina*. However, the prevailing conditions in the OTC apparently weakened *T. pisana* to such an extent that only few individuals survived another heat shock a week later. In contrast, *X. derbentina* exhibited heat hardening induced by the OTC conditions, meaning that a heat shock after a week of adaptation to the OTCs resulted in much lower mortality than in individuals of the same species that had not adapted to OTC conditions before the heat shock. After a week of exposure to OTCs, the survival rate following heat shock was also significantly higher in *X. derbentina* than in *T. pisana*. These results suggest that *X. derbentina* will have a selective advantage over *T. pisana* as their environment continues to warm up unless more heat-tolerant phenotypes of the latter species evolve or immigrate.

**Graphical Abstract:**

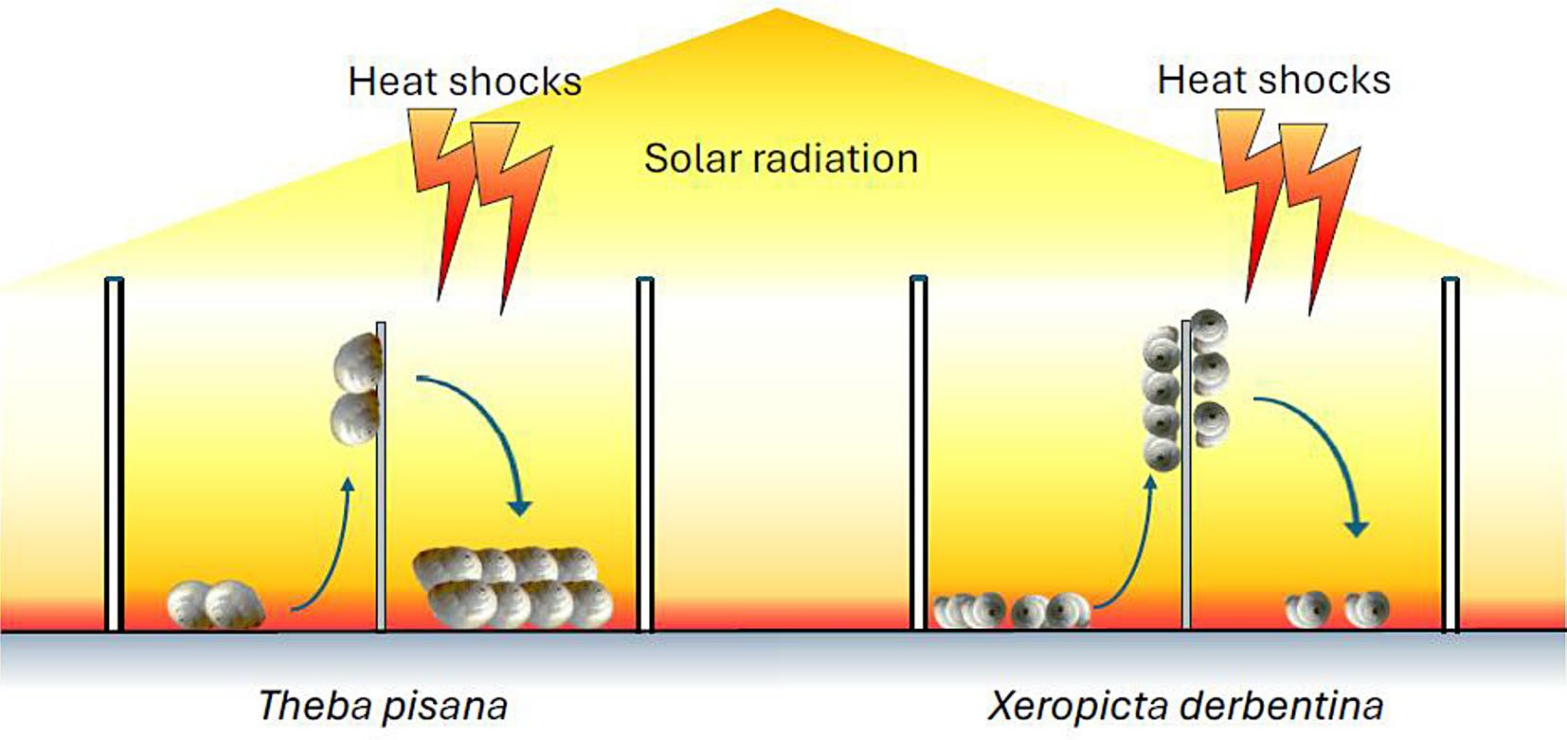

## Background

**Fig. 1:**
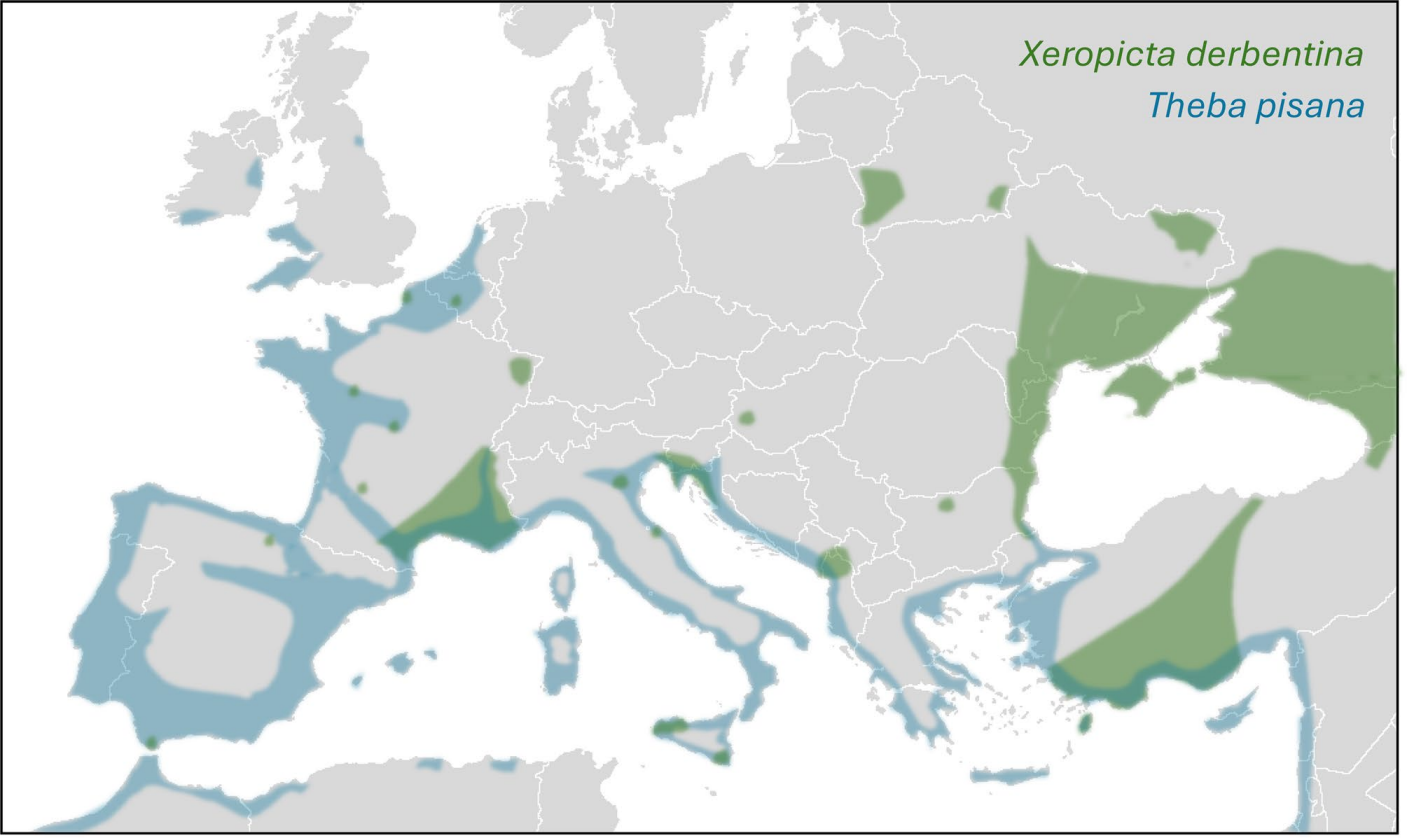
Distribution map of *T. pisana* (blue) and *X. derbentina* (green) in Europe and adjacent regions according to information obtained from [1, 2, 4–9] https://www.gbif.org/species/4564893 (global Biodiversity information Facility); and http://www.animalbase.uni-goettingen.de. These species occur sympatrically in stable populations in southern France, on the southern coast of Turkey, and in Istria

The Mediterranean region is home to a wide variety of helicoid snail species, some of which have distribution areas that cover large regions with very different climatic conditions [1] . In these habitats, snails can occur in extremely large abundance. Some species are highly heat-tolerant and can withstand direct sunlight all day for many weeks in an inactive stage. Two ecologically successful species with large ranges and widespread occurrence are the white garden snail, also known as the Mediterranean snail (*Theba pisana* (O.F. Müller, 1774), Helicidae), and *Xeropicta derbentina* (Krynicki, 1836) (Geomitridae). While *T. pisana* is considered a typical inhabitant of circum-Mediterranean ecosystems, *X. derbentina* was introduced to the southern French Provence region in the 1940s and has since become widespread there [2]. The primary distribution area of *T. pisana* extends from the British Isles and Ireland across western, southern and south-eastern Europe to North Africa and the Arabian Peninsula, including areas bordering the Red Sea – regions that are very hot and dry. The species has also been introduced to California, southern Africa, and Australia [3]. *Xeropicta derbentina* is also native to hot, dry habitats such as the steppes north of the Black Sea, the eastern Mediterranean region, Anatolia, and the Caucasus. However, in the last century, it has also become established as a neozoon in southern France, Ukraine, southwestern Russia [4] and, recently, Croatia, Italy and Belarus [5–7]. It was recorded in northeastern France [8, 9], and two live but isolated individuals were recently found in Belgium [10]. As *X. derbentina* is not usually found in humid coastal areas and dunes [1], the two species predominantly occur sympatrically in the inner part of Provence today (Fig. 1).

Due to their similar habitat requirements, *T. pisana* and *X. derbentina* may be potential competitors for environmental resources, particularly the most suitable places to stay, e.g. shady spots or objects to climb, in this area. Furthermore, it is expected that thermal pressure on both species will increase in the future due to global warming, as the circum-Mediterranean region is one of the areas most affected by it worldwide [11]. Due to thermal selection favouring light-coloured shells in areas with high solar radiation [12–14], both species in the open landscape of inland Provence exhibit a preference for a white, unpigmented shell phenotype. Both species naturally escape the hot soil’s surface by climbing vertical objects [14]. Furthermore, both species are considered to be highly thermotolerant in terms of this phenotype and their biochemical, physiological, and cellular capabilities for compensating for heat stress have been well characterised (*T. pisana* [15–18]: and [19, 20] and [21]*X. derbentina* [22]: and [23, 24] and [25, 26] and [27, 28]). Some studies have compared the sub-organismic reactions of the two species to heat directly [29–32]. These results indicate that *X. derbentina* is slightly more tolerant of constantly high ambient temperatures than *T. pisana*,in terms of the histological integrity of the hepatopancreas and the induction of stress proteins, at least after eight hours of artificial exposure in an incubator.

In addition to pigmentation, the structure of the shell could also be thermodynamically relevant. Infrared thermography has revealed that the temperature of inactive snails’ shells in direct sunlight can be much lower than that of the objects to which they are attached [33, 34]. In recent years, the effect of radiative cooling, i.e. the emission of energy at wavelengths that are barely absorbed by the Earth’s atmosphere has been described and demonstrated in Saharan silver ants, *Cataglyphis bombycina* [35, 36]. In these desert ants, this effect is based on hair-like microstructures on the body surface that are geometrically uniform, and it could be eliminated through the experimental modification of the insect cuticle [36]. Since the ostracum of the snail shell also consists of crystalline column fibres, radiative cooling could occur here too, possibly with species-specific intensity.

Thus, the coexistence of two morphologically similar and closely related species that both have very similar habitat requirements and large population densities provides an ideal model for addressing questions in evolutionary biology in the context of climate change. Specifically, we asked whether one of the two coexisting species have adaptive advantage as the habitat warms, and if so, which one.

To answer the following questions, we conducted field experiments with *T. pisana* and *X. derbentina* in the inner Provence in the summer of 2023, simulating global warming conditions in open-top chambers (OTCs) that increased the current prevailing temperatures by 1.15 °C [13]:

1. Do the surface temperatures of the shells of the two species differ in the OTCs?
2. Do the two species respond differently to an increase in ambient temperature in the OTCs in terms of survival?
3. Does interspecific competition for the best resting places further reduce the survival rate?
4. Are there any indications of radiative cooling effects when comparing natural individuals of both species with those whose shell surface structure has been altered by a clear varnish coating?
5. What role do heat shocks (caused by heat conduction from the hot soil surface) play in the survival of the two species?
6. Based on these findings regarding possible pre-adaptations of the species to climate change, what can be expected for the selection advantages of a particular species?

## Methods

### Field sites and open-top chamber experiments

**Fig. 2:**
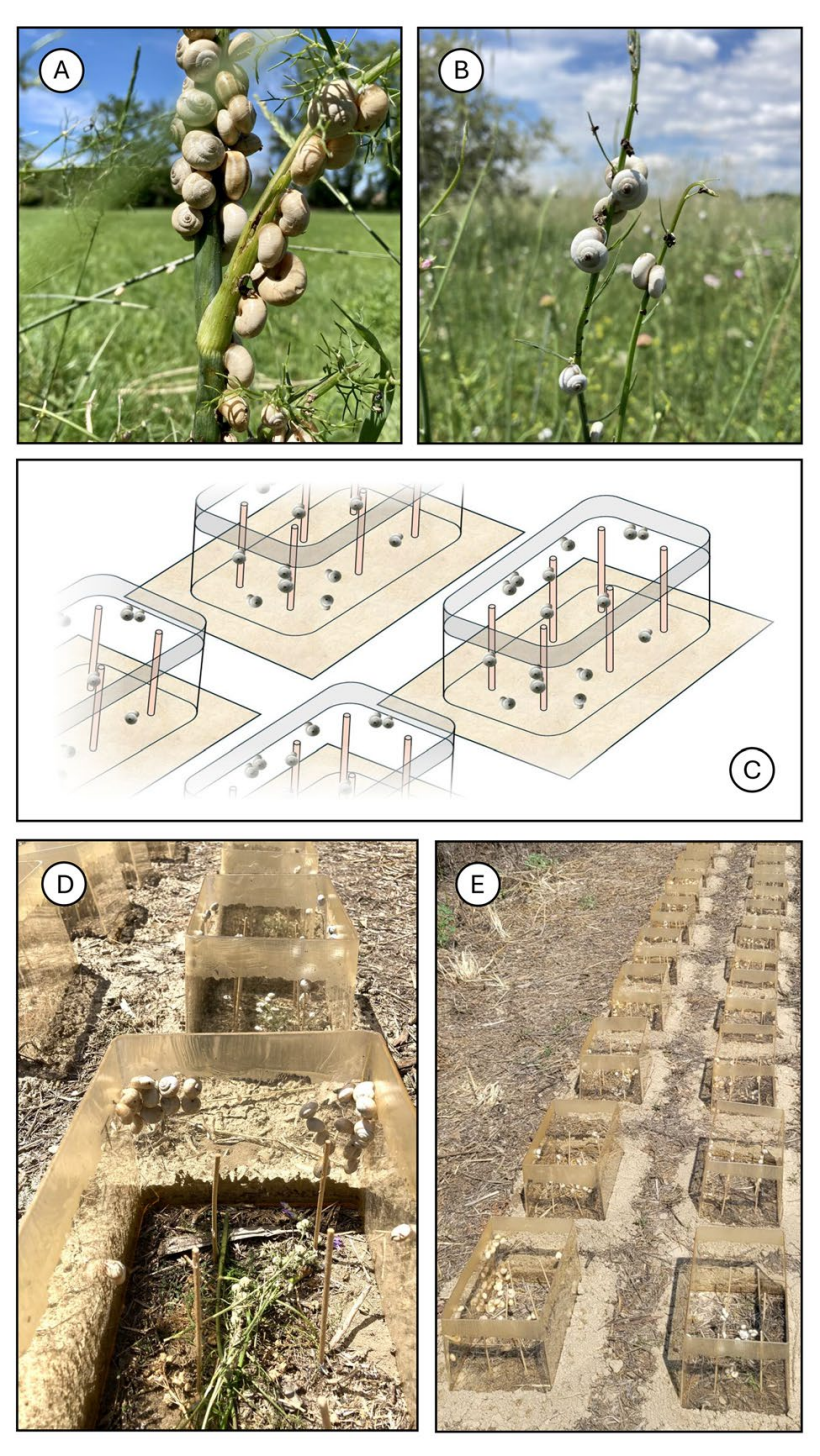
Habitus of the two helicoid land snail species in their natural habitats and exposure in open-top chambers (OTCs). **A**: *Theba pisana* (O. F. Müller, 1774) at the sampling site near Montfavet (43° 54.984´ N, 04° 53.772´ E). **B**: *Xeropicta derbentina* (Krynicki, 1836) at the sampling site near Mazan (44° 02.809´ N, 05° 07.590´ E), both dept. Vaucluse, Provence, France. **C**: schematic representation of OTCs with vertically positioned wooden skewers and lubricated upper rims. **D**: exposure of snails in the OTCs. **E**: OTCs arranged in two rows on unshaded ground at INRAE Avignon (43° 54.910´ N, 04° 52.861´ E)

**Fig. 3:**
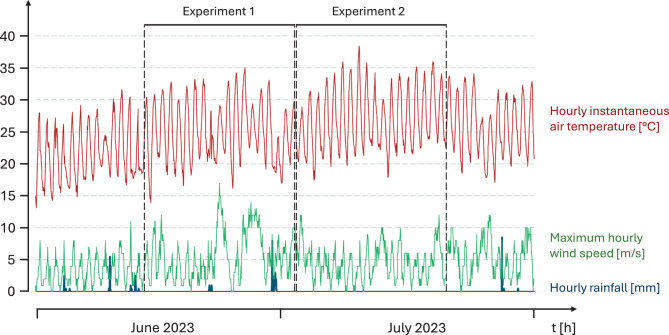
Climate data for the experimental site (Montfavet, southern France, 43.916857 N, 4.877207E) during the time of the global warming simulation experiments (14 June − 21 July 2023). Top: hourly instantaneous air temperature [°C] in the shadow at 2 m above ground as recorded in Avignon climate station, about 200 m away from the experimental site (red). Middle: maximum hourly wind speed [m/s] at the climate station (green). Bottom: precipitation measured as hourly rainfall [mm] at the climate station (blue). The scaling of the y-axis refers to all three parameters. Data recorded for June and July 2023; exposure periods for experiment 1 (June 14 – July 2, 2023) and experiment 2 (July 2 – July 21, 2023) are depicted. Mean daytime (9–21 hr) air temperature outside the chambers during experiment 1: 28.2 ± 3.5 °C (maximum temperature: 35.0 °C); during experiment 2: 30.3 ± 3.2 °C (maximum temperature: 38.4 °C)

We selected the Avignon area of southern France for our experiments. Random sampling of adult *Theba pisana* (O. F. Müller, 1774) took place at a site close to Montfavet (43° 54.984´ N, 04° 53.772´ E), while adult *Xeropicta derbentina* (Krynicki, 1836) were sampled from a site near Mazan, on the Bon Remède territory (44° 02.809´ N, 05° 07.590´ E) (Fig. 2A, B). Both sampling events took place on 11 June 2023 and both sites were densely populated with these snails. They also were in the vicinity of the Avignon climate station. From this station we obtained climate data (hourly instantaneous air temperature at 1 m above ground level in the shade [°C], maximum hourly wind speed [m/s] and precipitation, measured as hourly rainfall [mm], see Fig. 3) for the period of our experiments (14 June − 21 July 2023; temporal resolution: 1 h). To ensure equal starting conditions, both species were initially kept under laboratory conditions (25 °C and a natural day/night cycle for a minimum of 2d) for all experiments.

Passive open-top chambers (OTCs) were constructed to locally raise the temperature in the snails’ habitat and simulate environmental warming conditions [37, 38]. We used the same construction method for the OTCs as that which has previously been shown to increase the mean temperature at a distance of 5 cm above ground level by an average of 1.12 °C compared to outside temperatures (Fig. 2C). This is considered a realistic simulation of global warming in the Avignon area, where the monthly mean temperature has increased by 1.34 °C over the past 30 years [13].

We conducted two experiments. The first experiment took place from 14 June to 2 July 2023. Fifty transparent polyethylene OTCs, each with a ground area of 30 × 20 cm and a height of 20 cm, were arranged in two rows of 25, with 20 cm between them. The OTCs were positioned on the ground in a freshly mown area of natural grassland on the grounds of INRAE Avignon (43° 54.910´ N, 04° 52.861´ E), 200 m from the Avignon climate station (Fig. 2D, E). They were fixed to the ground with wire and spikes. The experimental area measured 10 × 1 m and was level and shadeless. The second experiment in the same field plot, which involved 20 OTCs only, lasted from 2 July to 21 July 2023. Prior to the experiments, all abundant land snails were removed from the experimental area. To provide climbing facilities for the snails, two rows of three wooden skewers were vertically inserted into the ground in each OTC, leaving 20 cm above the ground surface. To prevent snails from escaping from the experimental setup, the upper rim of each OTC was lubricated with a paste containing lemon extract (IRKA Meitingen, Germany), which repels snails but has been proven not to cause mortality [13]. Shoots and leaves of fennel (*Foeniculum vulgare*) and common mallow (*Malva sylvestris*), i.e. usual food plants for both species, were provided in more than sufficient quantity and in a fresh state in both experiments and served *ad libitum* as a food source. Any dietary restrictions can be disregarded. Among the OTCs, there were no losses of experimental animals, such as those that could occur due to escape or predation.

### Experimental design

The experiments were designed to investigate (i) the survival of individual snails, as indicated by their climbing of skewers or OTC walls, and (ii) the shell surface temperature of snails that had climbed (subsequently referred to as ‘up’). In one group of the field-collected snails, the entire surface of the shell was coated with a transparent clear varnish to alter the micro- and nanostructures of the shell on its surface and thus eliminate or mitigate any potential radiative cooling effects. Five groups of snails were set up: (i) natural *T. pisana* kept separately from *X. derbentina* in groups of 40 individuals; (ii) natural *X. derbentina* kept separately from *T. pisana* in groups of 40 individuals; (iii) a mixture of natural *T. pisana* and *X. derbentina*, with 20 individuals of each species competing for climbing positions in the same OTC; (iv) varnished *T. pisana* kept separately from *X. derbentina* in groups of 40 individuals; and (v) varnished *X. derbentina* kept separately from *T. pisana* in groups of 40 individuals. For each of these groups, we set up ten replicate OTCs in the first experiment, the spatial order of which was random. In this experiment, we not only exposed the animals to the increased ambient temperatures in the OTCs for 18 days but also applied two heat shocks by placing the animals singularly on the hot ground in the OTCs at 4 o´clock in the afternoon on day 0 (i.e. without prior acclimatization to the OTCs) and at 11 o´clock in the late morning on day 7 (i.e. at temperatures equal to the first heat shock after a week of OTC acclimatization/exposure). As measured by thermography, the mean soil surface temperature (± standard deviation) in the OTCs was 68.1 ± 5.5 °C (*n* = 50) from late morning to afternoon.

As the results for the varnished and competing snails were similar to those for the natural snails suggesting negligible effects of these parameters, in the second experiment we limited the number of snail groups investigated to (I) natural *T. pisana*, kept in groups of 40 individuals and separated from *X. derbentina*, and (II) natural *X. derbentina*, kept in groups of 40 individuals and separated from *T. pisana*. The number of OTC replicates remained at 10. In the second experiment, the snails were not heat-shocked initially but were only subjected to a heat shock after a week of OTC acclimatization/exposure by being singularly placed on the hot ground. For both experiments, ‘control’ snails were kept in three groups of 40 individuals in the laboratory under constant conditions of temperature (25 °C) and a natural day/night cycle. These laboratory-kept snails were not intended to mimic field conditions, but rather to ensure the safety of the containers and repellent paste used. As it was impossible to keep ‘control’ snails in a reasonably sized outdoor cage without affecting the temperature, we decided to relate the survival rate observed in our experiments to that of free-living, non-heat-shocked *T. pisana* individuals that climbed vertically in the Montfavet area in summer. This survival rate was 95.5% [13].

### Determination of survival rate and shell temperature

As it is virtually impossible to tell whether an inactive snail is alive or dead with the naked eye, the position of the animal (whether it had climbed ‘up’) was considered an indicator of survival. Individuals lying on the ground were considered dead. In the first experiment, we checked the survival rate at 0, 1, 2, 6, 9, 14 and 18 days of exposure. In the second experiment, we checked the survival rate at 0, 1, 2, 4, 5, 9, 14 and 18 days of exposure. The ‘up’ snails´ shell surface temperature was measured by thermography using a high-resolution thermal imaging camera (Micro-Epsilon thermo IMAGER TIM 450 equipped with a macro lens) and the TIM Connect software (Micro-Epsilon, Ortenburg, Germany). The temperature of the shells was only determined for inactive individuals at a height of at least 10 cm above the ground, either on the inside of the OTCs or on wooden skewers, and only for the side opposite to the ground and facing the sun, measured perpendicular from above.

### Statistics

Data were checked for homoscedasticity (homogeneity of variance) using Levene´s test and for normal distribution using the Shapiro-Wilk test. As both criteria were not always met, the significance of the pairwise comparisons of the data on the survival rates of *T. pisana vs. X. derbentina*, which were recorded on the very same day in each case, was checked using the non-parametrical Kruskal-Wallis test. We did not conduct comparisons across days but nevertheless corrected the *p* values for multiple comparisons according to the Benjamini-Hochberg method because survival data across days were not independent from one another. Homogeneity of variance and normality in the thermometric data were also checked using Levene´s test and the Shapiro-Wilk test. As homoscedasticity was not met, significance was checked using the Kruskal-Wallis test. Significance levels were set at *p* > 0.05 (not significant), 0.05 ≥ *p* > 0.01 (slightly significant), 0.01 ≥ *p* > 0.001 (significant) and *p* ≤ 0.001 (highly significant). All statistical work was carried out using SAS JMP 18.2.1 software.

## Results

### Thermography

**Fig. 4:**
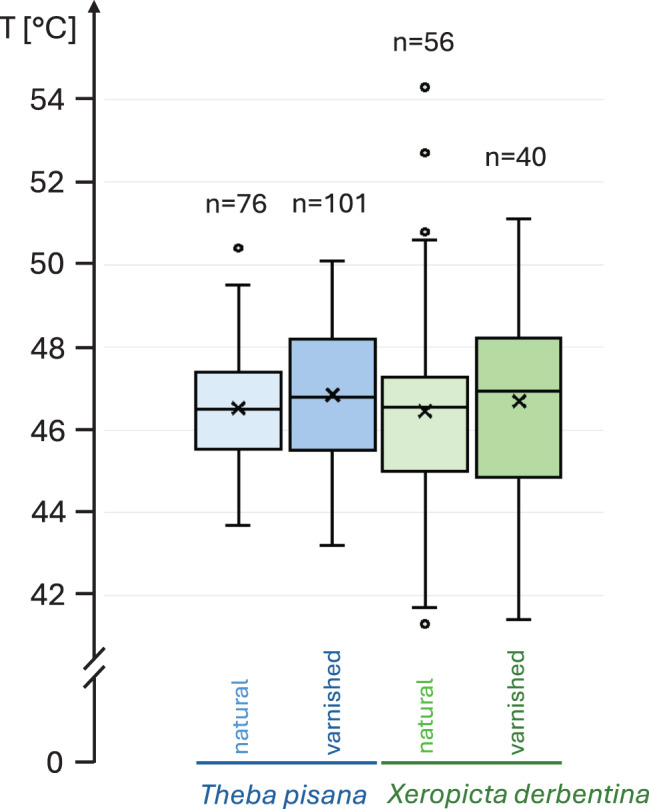
Shell surface temperatures of sunlight-exposed *T. pisana* (blue) and *X. derbentina* (green) residing in the OTCs, measured by thermography. Only individuals that have climbed objects rising above the soil surface were considered. Box plots refer to 25, 50 and 75% percentiles. The whiskers extend to the smallest or largest non-outlier value. Crosses: arithmetic means; small circles: outliers. Data lack significant difference (Levene´s test: no homogeneity of variance, Kruskal-Wallis test: *p* = 0.3339)

The shell surface temperature, measured from above by thermography, hardly differed between the two species, and between the natural shells and those coated with a transparent varnish (Fig. 4). *T. pisana* had an average surface temperature of 48.525 ± 1.340 °C (*n* = 76) and was only insignificantly (0.068 °C) warmer than *X. derbentina* with an average temperature of 48.457 ± 2.478 °C (*n* = 56). Varnish coating insignificantly increased the surface temperature of *T. pisana* slightly by 0.327 °C to 48.852 ± 1.655 °C (*n* = 101) and of *X. derbentina* by 0.246 °C to 48.703 ± 2.209 °C (*n* = 40).

### Survival

In all cases, the survival rate of laboratory-kept snails at 25 °C over 18 days was very high (*T. pisana*: 94.2–96.7%; *X. derbentina*: 86.7–89.2%). However, significant differences in survival (assessed using the proxy ‘up’ position) were observed between the two species in OTC-exposed individuals depending on the duration of exposure and the number of acute heat shocks.

**Fig. 5:**
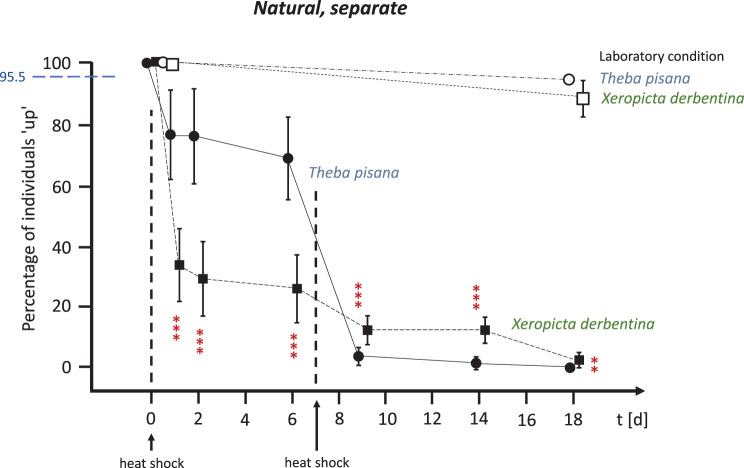
Survival rates of natural, separately kept *T. pisana* (circles) and *X. derbentina* (squares) over an 18-day period. Percentage of individuals that climbed (‘up’) vs. exposure time in days. Open symbols represent laboratory-kept snails and filled symbols represent snails in field-installed OTCs. Heat shocks (direct single positioning on the ground) were applied on days 0 and 7. Means ± SD. The dashed blue horizontal line represents the survival rate of *T. pisana* at the Montfavet site under current climatic conditions in summer 2017 without experimental heat shock (2063 survivors of 2160 randomly collected adult individuals climbed ‘up’ in the field [13]). Significance of data obtained for *T. pisana vs. X derbentina* (*** highly significant, ** significant) at *t* = 1d: *p* = 0.0005; both 2d and 6d: *p* = 0.0004; 9d: *p* = 0.0005; 14d: *p* = 0.0006; and 18d: *p* = 0.0018 (Kruskal-Wallis test, Benjamini-Hochberg-corrected)

In the first experiment, natural (non-varnished) individuals kept separately in OTCs (see Fig. 5) experienced a sharp decline in survival following the initial heat shock at the start of the experiment on day 0, without prior acclimatisation to OTC conditions. *T. pisana* had a survival rate of 76.5% on day 1; *X. derbentina* had a survival rate of only 33.8%. These values remained almost constant at 68.8–76.0% (*T. pisana*) and 26.0–29.3% (*X. derbentina*), respectively, over the following week of continuous exposure in the OTCs. Following a second heat shock on day 7, *T. pisana* ‘s survival rate on day 9 was just 3.8%, compared to 12.5% for *X. derbentina*. These values remained almost constant over the next five days. After 18 days, no *T. pisana* were still alive, and only a few *X. derbentina* remained.

**Fig. 6:**
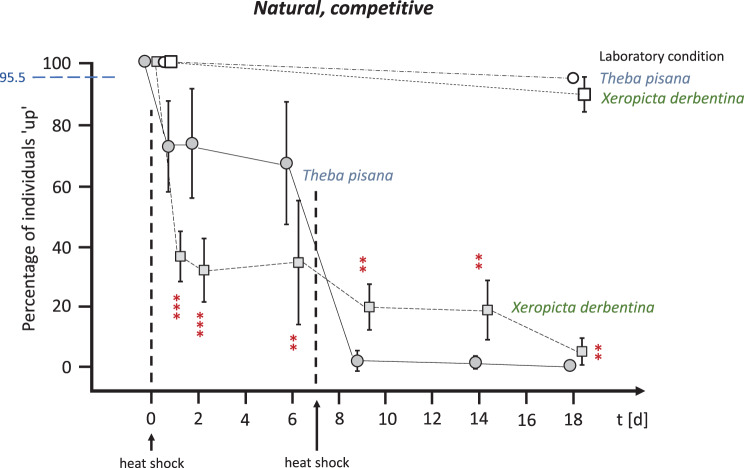
Survival rates of natural *T. pisana* (circles) and *X. derbentina* (squares) exposed jointly and thus competing in the OTCs over an 18-day period. Percentage of individuals that climbed (‘up’) vs. exposure time in days. Open symbols represent laboratory-kept snails and shaded symbols represent snails in the field-installed OTCs. Heat shocks (direct single positioning on the ground) were applied on days 0 and 7. Means ± SD. Dashed blue horizontal line as in Fig. 5. Significance of data obtained for *T. pisana vs. X derbentina* (*** highly significant, ** significant) at both *t* = 1d and 2d: *p* = 0.0009; 6d: *p* = 0.0050; both 9d and 14d: *p* = 0.0012; and 18d: *p* = 0.0018 (Kruskal-Wallis test, Benjamini-Hochberg-corrected)

**Fig. 7:**
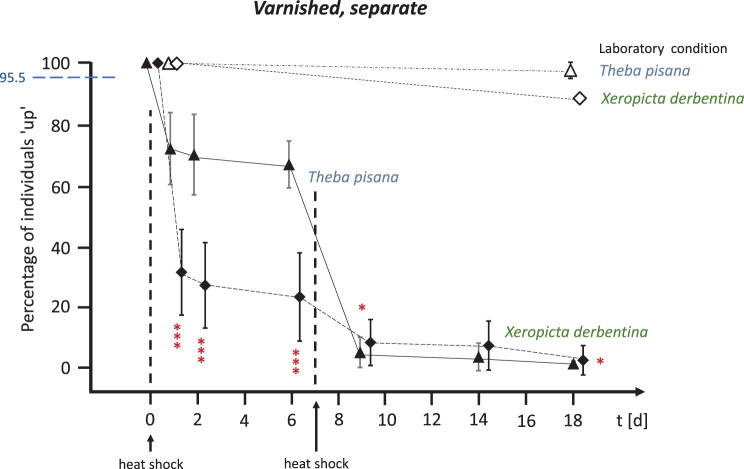
Survival rates of separately kept *T. pisana* (triangles) and *X. derbentina* (diamonds) with varnished shell surface over an 18-day period. Percentage of individuals that climbed (‘up’) vs. exposure time in days. Open symbols represent laboratory-kept snails and filled symbols represent snails in field-installed OTCs. Heat shocks (direct single positioning on the ground) were applied on days 0 and 7. Means ± SD. Dashed blue horizontal line as in Fig. 5. Significance of data obtained for *T. pisana vs. X derbentina* (*** highly significant, * slightly significant) at *t* = 1d, 2d and 6d: *p* = 0.0006; 9d: *p* = 0.0493; 14d: *p* = 0.0900; and 18d: *p* = 0.0244 (Kruskal-Wallis test, Benjamini-Hochberg-corrected)

When the two species were kept together in OTCs (‘competitive’, see Fig. 6) or when their shells were coated with clear varnish (‘varnished’, see Fig. 7), the survival rate was similar to that of the isolated natural individuals. Following the initial heat shock, 72.0% of *T. pisana* (competitive) and 71.5% of *T. pisana* (varnished) individuals survived on day 1, compared to 36.5% of *X. derbentina* (competitive) and 32.3% of *X. derbentina* (varnished) individuals. After a second heat shock one week later, only 20.0% (competitive) and 9.5% (varnished) of *X. derbentina* were still alive on day 9, compared to 1.5% (competitive) and 4.0% (varnished) of *T. pisana*. Complete mortality was observed for *Theba* after 18 days and almost complete mortality for *Xeropicta* in all cases.

**Fig. 8:**
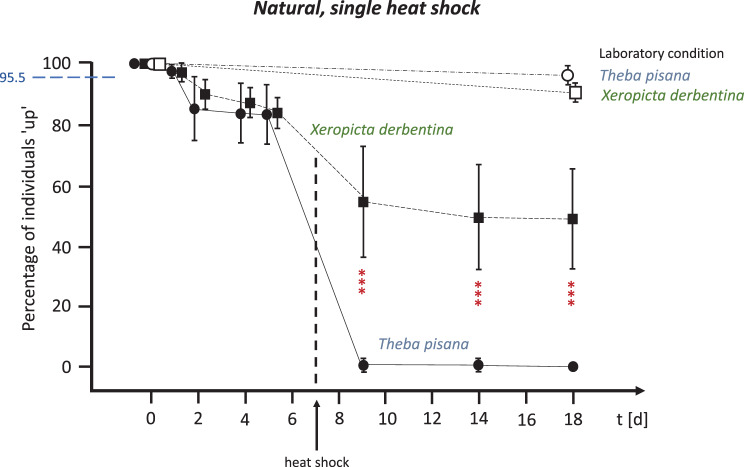
Survival rates of natural, separately kept *T. pisana* (circles) and *X. derbentina* (squares) over an 18-day period. Percentage of individuals that climbed ‘up’ vs. exposure time in days. Open symbols represent laboratory conditions and filled symbols represent snails in field-installed OTCs. A single heat shock (direct single positioning on the ground) was applied on day 7. Means ± SD. Dashed blue horizontal line as in Fig. 5. Significance of data obtained for *T. pisana vs. X derbentina* (*** highly significant) at *t* = 1d: *p* = 0.3225; 2d: *p* = 0.5145; 4d: *p* = 0.7896; 6d: *p* = 0.7874; both 9d and 14d: *p* < 0.0002; and 18d: *p* < 0.0004 (Kruskal-Wallis test, Benjamini-Hochberg-corrected)

When the field-collected snails were given a week to acclimatise to the OTCs prior to the initial (and sole) heat shock in the second experiment, the outcome changed considerably (see Fig. 8). During this period, both species survived equally well, with survival rates never falling below 84.0% (*T. pisana*) and 85.0% (*X. derbentina*). However, the two species responded to the heat shock on day 7 in completely different ways. While *T. pisana*‘s survival rate dropped dramatically to 1.0% by day 9, with all individuals dead by day 18, 55.0% of *X. derbentina* survived this heat shock. Even after 18 days, almost half (49.5%) of the *X. derbentina* individuals were still alive.

## Discussion

Our study revealed significant differences in survival rates between *T. pisana* and *X. derbentina* under permanently elevated ambient temperatures and after a sudden heat shock, which mimics the situation in which individuals are stripped from their resting position by larger animals, agricultural machinery or humans, as well as by strong winds bending the plants on which they are sitting to the ground. However, no effect of direct competition between the two species could be demonstrated; the survival rate patterns essentially did not differ from those of species kept separately. No effect of varnishing was observed either: the pattern of survival rates and the surface temperatures of the shells were very similar to those of natural, non-varnished specimens. Although the temperature of varnished shells was minimally and insignificantly higher than that of natural shells in both species, it is questionable whether this extremely small and insignificant effect size indicates the importance and even presence of radiative cooling in these animals.

As the shell temperatures of *T. pisana* and *X. derbentina* did not differ in the OTCs, the observed differences in survival rates must be due to parameters other than the absorption and emission of solar radiation. Possible explanations include differences in size, shell thickness and physiological and biochemical responses to heat stress. Clearly, both species employ different strategies in this regard.

**Fig. 9:**
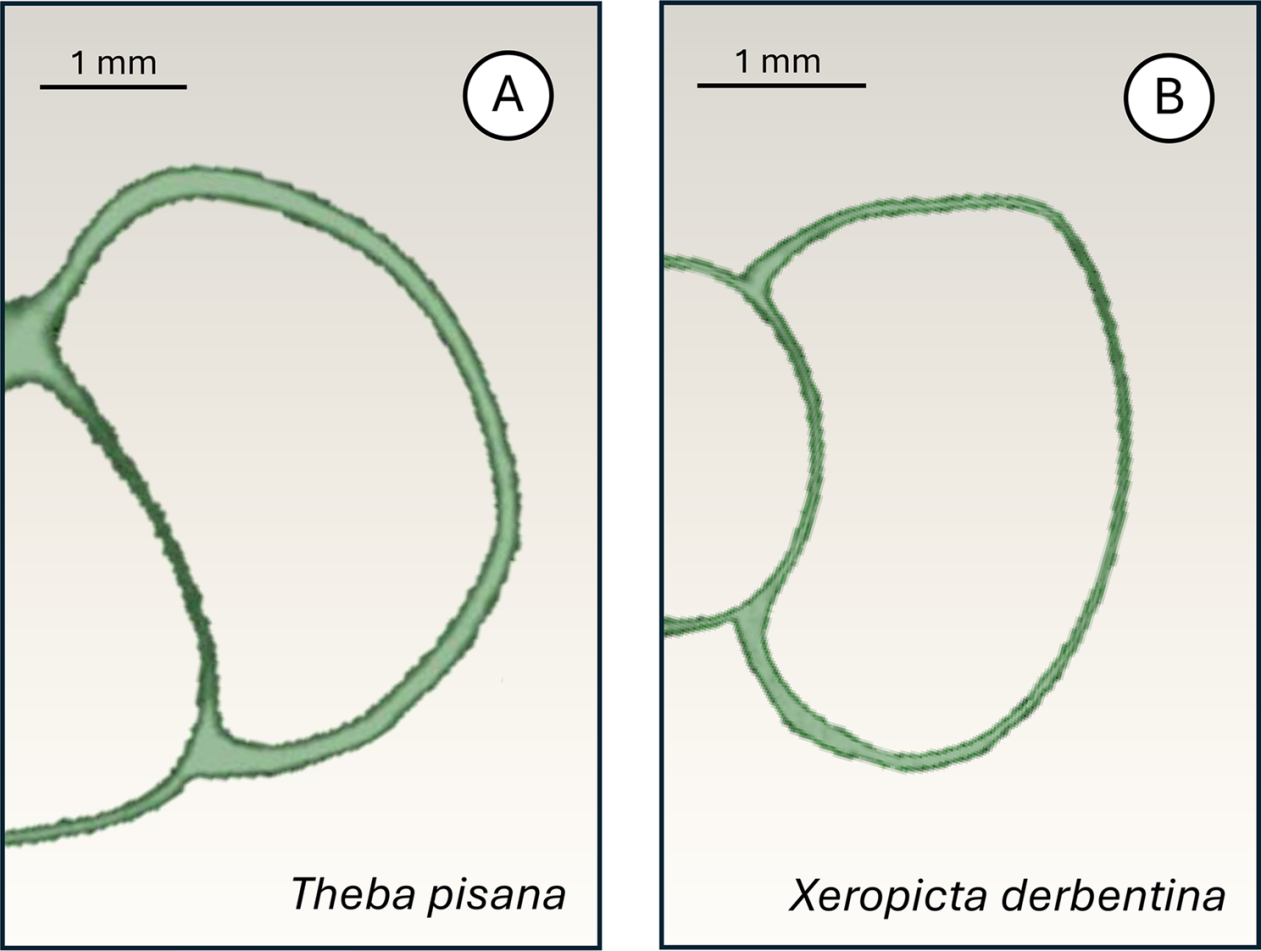
Micro-computer tomography (µCT) of the outer whorl of juvenile shells of *T. pisana* (**A**) and *X. derbentina* (**B**). Pictures taken from unpublished work of Christina Kyriakouli, Gabriel Ferreira, Helen Gabel, Sascha Zimmermann and Heinz-R. Köhler, all Tübingen University. The difference in the wall thickness of the shells between the two species is clearly visible in parts that are in direct contact with the environment

*Theba pisana* proved to be more tolerant than *X. derbentina* in response to acute heat shock caused by conduction. Its larger size (maximum diameter 25 mm [1], 18.8 mm according to own data from the Montfavet population) compared to *X. derbentina* (maximum diameter 20 mm [1], 13.4 mm according to own data from the Mazan population) gives it a lower surface-to-volume ratio. This suggests that the larger soft body of *T. pisana* heats up more slowly when the shell comes into contact with a hot surface, giving the animal more time to react and escape than the smaller *X. derbentina*. Additionally, *X. derbentina*‘s lower shell thickness, compared to *T. pisana* (Fig. 9), favours faster heat conduction from the shell surface to the soft body which further limits the chances of escaping protein-denaturing temperatures in time.

Even within the *T. pisana* species, there are differences in shell thickness between individuals, with thicker shells found in habitats with higher aridity [39]. This therefore indicates that thicker shells are an adaptation to high summer temperatures and low water supply.

By contrast, *T. pisana* proved to be more sensitive than *X. derbentina* to continuously elevated temperatures in the OTCs. In a very similar study conducted in the same area in 2017, 79.7% of unpigmented *T. pisana* individuals survived the conditions in OTCs of an identical design during the first seven days of exposure, even without additional heat shock. This finding is confirmed by our current data (Fig. 8). After 19 days, only 21.7% of these animals were still alive [13].

*Xeropicta derbentina* was highly sensitive to an unprepared heat shock; however, its tolerance of the temperatures permanently prevailing in the OTCs led to heat hardening, significantly increasing the survival rate following a subsequent heat shock. While only around a third of *X. derbentina* individuals survived an unprepared heat shock, the survival rate following one or more heat shocks after a week of heat hardening in the OTCs was twice this value, at around two thirds. By contrast, the same one-week exposure in the OTCs weakened *T. pisana* individuals so severely that only a few specimens survived the subsequent heat shock. This induced heat hardening is most likely due to *X. derbentina*‘s high capacity to synthesise heat shock proteins, which stabilise the correct folding of intracellular proteins at high temperatures [40]. A study by Köhler et al. [30] comparing the two species showed that the maximum induction of Hsp70 stress proteins in *X. derbentina* occurred after eight hours of exposure at 43–48 °C (depending on the population), whereas in *T. pisana* it occurred at 38 °C and decreased at higher temperatures, indicating an overload of this biochemical protection system. *X. derbentina* was also able to increase the baseline level of Hsp70 by up to sixfold after eight hours of heat exposure, whereas *T. pisana* increased the Hsp70 level by only 3.35-fold [30]. These differences in the capacity of the Hsp70 system may explain the effects on mortality documented in the current study and the heat hardening observed in *X. derbentina*.

In terms of cellular integrity, *T. pisana* has been described to be less tolerant of high temperatures than *X. derbentina*. After eight hours of exposure to 45 °C, digestive cells (and the overall histological condition of the midgut gland) were severely affected in *T. pisana*, whereas in *X. derbentina* the effect was much less pronounced [29]. The percentage of calcium cells in this organ was also significantly lower in *T. pisana* (approx. 35%) than in *X. derbentina* (approx. 80%), reducing *T. pisana*‘s capacity to maintain the internal acid-base balance that is potentially disturbed by high temperatures [41, 42]. These effects have resulted in 50% of individuals becoming immobile after 8 hours at 42 °C in *T. pisana*, compared to 48–51 °C in *X. derbentina* [29]. *Xeropicta derbentina* is also much more capable of recovering from heat-induced stress during a recovery phase, a trait that has been attributed to its ability to induce hypertrophy and hyperplasia of calcium cells, enabling it to cope with heat-induced osmotic stress [31]. Therefore, from a biochemical and cellular biology perspective, it is justifiable that *T. pisana* is not as resistant to long-term, constantly elevated temperatures as *X. derbentina*.

Our study revealed two distinct thermotolerance strategies in these two species. While *T. pisana* is better able to cope with short-term thermal stress caused by heat conduction because of its body size and shell thickness, *X. derbentina*, however, is better able to withstand continuously elevated temperatures over the long term because of more effective and stable inducible biochemical and cellular processes at higher temperatures. As both species, like many helicoid snails in hot and dry areas, exhibit pronounced climbing behaviour, which removes them from the hot substrate and thus avoids heat conduction, the progressive continuous warming of the habitat in the course of global change will be the more decisive thermal selection factor. The *X. derbentina* phenotypes currently found in Provence can definitely cope better with this stressor than the *T. pisana* phenotypes can, and so they may potentially replace *T. pisana* in parts of this area in the future. However, it is also conceivable that increased thermal pressure could lead to the selection of more temperature-tolerant or plastic *T. pisana* phenotypes, or that phenotypes from hotter North African areas could become established. However, even this does not guarantee population survival due to possible fitness costs related to trade-offs [43]. Also, the upper thermal limit is particularly rigid in heat-loving species, leaving little room for further evolutionary adaptations and making these species highly vulnerable to global warming [44]. Furthermore, genetic adaptation driven by climate change has only been proven beyond doubt in a few cases to date, e.g. in water fleas (*Daphnia magna* [45]) and field mustard (*Brassica rapa* [46]). Therefore, it is highly unlikely that spontaneous mutations leading to greater heat tolerance will occur, or that the genetic material of sporadically displaced *Theba* individuals from North Africa will prevail in populations consisting of tens or hundreds of thousands of individuals. Thus, in this respect, we speculate that, in our case, the neobiotic species *X. derbentina* may prevail in the future in some areas of the Mediterranean that continue to warm where both species currently occur sympatrically. The Theba Survey Consortium’s (unpublished) ongoing habitat suitability modelling, based on multiple abiotic and biotic environmental factors, predicts deteriorating conditions in a number of Mediterranean regions for *T. pisana*, with a high likelihood of extinction there. It remains to be seen whether *X. derbentina* can endure these shifts in conditions in the long term.

## Data Availability

The datasets and analyses of this study are available from the corresponding author on reasonable request.

## References

[1] Welter-Schultes FW. European non-marine molluscs, a guide for species identification. Göttingen: Planet Poster Editions; 2012.

[2] Kiss L, Labaune C, Magnin F, Aubry S. Plasticity of the life cycle of *Xeropicta derbentina* (Krynicki, 1836), a recently introduced snail in mediterranean France. J Molluscan Stud. 2005;71(3):221–31. 10.1093/mollus/eyi030.

[3] EPPO (European and Mediterranean Plant Protection Organization). *Theba pisana* (THEBPI). In: Global Database. Paris: France. Last update 2023. 18 Dec 2025. https://gd.eppo.int/taxon/THEBPI/distribution.

[4] Adamova VV, Orlov MA. Land snails *brephulopsis cylindrica* and *Xeropicta derbentina* (Gastropoda: stylommatophora): case study of invasive species distribution modelling. Ruthenica, Russ Malacological J. 2022;32(3):121–36. 10.35885/ruthenica.2022.32(3).5.

[5] De Mattia W. *Xeropicta derbentina* (Krynicky, 1836) (Gastropoda, Hygromiidae) in Italy and along the Croatian coast, with notes on its systematics and nomenclature. Basteria. 2007;71:1–12.

[6] De Mattia W, Pesic V. *Xeropicta* (Gastropoda, Hygromiidae) goes west: the first record of *X. krynickii* (Krynicki, 1833) for Montenegro, with a description of its shell and genital morphology, and an additional record of *X. derbentina* (Krynicki, 1836) for Italy. Ecol Mont. 2014;1(4):193–200. 10.37828/em.2014.1.27.

[7] Ostrovsky AM. *Xeropicta derbentina* (Krynicki, 1836) (Gastropoda: Eupulmonata: geomitridae) in Belarus – new data. Folia Malacol. 2023;31(1):43–47. 10.12657/folmal.031.006.

[8] Bartolucci JC, Bertrand A. Première observation de *Xeropicta derbentina* (Krynicki, 1836) (Mollusca, Gastropoda, Geomitridae), en Haute-Marne (France, Grand Est). Folia Conchyliologica. 2022;66:73–76.

[9] Wagner A. Première observation de l’Hélicelle des Balkans, *Xeropicta derbentina* (Krynicki, 1836) (Mollusca, Gastropoda, Geomitridae) en Alsace (France, Grand Est, Bas-Rhin). Bull de la Société d’Histoire naturelle et d’Ethnographie de Colmar. 2021;77:144–45.

[10] Bronne L, Lassman M. First interception of *Xeropicta derbentina* in the Benelux (Gastropoda: geomitridae). J Conch. 2026;45(5):695–700. 10.61733/jconch/4561.

[11] , Lee, H, Romero, J, editors.IPCC (Intergovernmental Panel on Climate Change). Summary for Policymakers. in: Writing. Climate change, 2023: synthesis Report. Contribution of working groups I, II and III to the sixth assessment Report of the Intergovernmental Panel on climate change. Geneva: IPCC; 2023. p. 1–34. 10.59327/IPCC/AR6-9789291691647.001.

[12] Hazel WN, Johnson MS. Microhabitat choice and polymorphism in the land snail *Theba pisana* (müller). (Müller) Heredity. 1990;65(3):449–54. 10.1038/hdy.1990.116.

[13] Köhler H-R, Capowiez Y, Mazzia C, Eckstein H, Kaczmarek N, Bilton MC, et al. Experimental simulation of environmental warming selects against pigmented morphs of land snails. Ecol Evol. 2021;11(3):1111–30. 10.1002/ece3.7002. 33598118 10.1002/ece3.7002 PMC7863387

[14] Schweizer M, Triebskorn R, Köhler H-R. Snails in the sun: strategies of terrestrial gastropods to cope with hot and dry conditions. Ecol Evol. 2019;9(22):12940–60. 10.1002/ece3.5607. 31788227 10.1002/ece3.5607 PMC6875674

[15] Arad Z. DESICCATION AND REHYDRATION IN LAND SNAILS?A TEST FOR DISTINCT SET POINTS IN *Theba pisana*. Isr J Zool. 2001;47(1):41–53. 10.1092/7N2K-U92C-0QGE-0QRP.

[16] Bose U, Centurion E, Hodson MP, Shaw PN, Storey KB, Cummins SF. Global metabolite analysis of the land snail *Theba pisana* hemolymph during active and aestivated states. Comp Biochem Physiol Part D Genomics Proteomics. 2016;19:25–33. 10.1016/j.cbd.2016.05.004. 27318654 10.1016/j.cbd.2016.05.004

[17] Mizrahi T, Goldenberg S, Heller J, Arad Z. Natural variation in resistance to desiccation and heat shock protein expression in the land snail *Theba pisana* along a climatic gradient. Physiol Biochem Zool. 2015;88(1):66–80. 10.1086/679485. 25590594 10.1086/679485

[18] Scheil AE, Scheil V, Triebskorn R, Capowiez Y, Mazzia C, Köhler HR. Shell colouration and antioxidant defence capacity in *Theba pisana*. Molluscan Research. 2012;32:132–6. 10.11646/mr.32.3.2.

[19] Mizrahi T, Goldenberg S, Heller J, Arad Z. Geographic variation in thermal tolerance and strategies of heat shock protein expression in the land snail *Theba pisana* in relation to genetic structure. Cell Stress Chaperones. 2016;21(2):219–38. 10.1007/s12192-015-0652-6. 26503612 10.1007/s12192-015-0652-6 PMC4786534

[20] Zimmermann S, Gärtner U, Ferreira GS, Köhler H-R, Wharam D. Thermal impact and the relevance of body size and activity on the oxygen consumption of a terrestrial snail, *Theba pisana* (Helicidae) at high ambient temperatures. Animals. 2024;14(2):261. 10.3390/ani14020261. 38254430 10.3390/ani14020261 PMC10812721

[21] Zimmermann S, Gärtner U, Capowiez Y, Köhler H-R, Wharam D. Water evaporation as a function of temperature, humidity, air velocity and body size in inactive terrestrial pulmonate *Theba pisana*. BMC Zool. 2025;10(1):13. 10.1186/s40850-025-00236-0. 40624592 10.1186/s40850-025-00236-0 PMC12232781

[22] Di Lellis MA, S SMT, Troschinski S, Mazzia C, Capowiez Y, Triebskorn R, et al. Solar radiation stress in climbing snails: behavioural and intrinsic features define the Hsp70 level in natural populations of *Xeropicta derbentina* (Pulmonata). (Pulmonata) Cell Stress Chaperones. 2012;17(6):717–27. 10.1007/s12192-012-0344-4. 22639082 10.1007/s12192-012-0344-4 PMC3468672

[23] Di Lellis MA, Sereda S, Geißler A, Picot A, Arnold P, Lang S, et al. Phenotypic diversity, population structure and stress protein-based capacitoring in populations of *Xeropicta derbentina*, a heat-tolerant land snail species. Cell Stress Chaperones. 2014;19(6):791–800. 10.1007/s12192-014-0503-x. 24609822 10.1007/s12192-014-0503-x PMC4389839

[24] Dieterich A, Fischbach U, Ludwig M, Di Lellis MA, Troschinski S, Gärtner U, et al. Daily and seasonal changes in heat exposure and the Hsp70 level of individuals from a field population of *Xeropicta derbentina* (Krynicki 1836) (Pulmonata, Hygromiidae) in southern France. Cell Stress Chaperones. 2013;18(4):405–14. 10.1007/s12192-012-0393-8. 23250584 10.1007/s12192-012-0393-8 PMC3682011

[25] Dieterich A, Troschinski S, Schwarz S, Di Lellis MA, Henneberg A, Fischbach U, et al. Hsp70 and lipid peroxide levels following heat stress in *Xeropicta derbentina* (Krynicki 1836) (Gastropoda, Pulmonata) with regard to different colour morphs. Cell Stress Chaperones. 2015;20(1):159–68. 10.1007/s12192-014-0534-3. 25108358 10.1007/s12192-014-0534-3 PMC4255243

[26] Troschinski S, Di Lellis MA, Sereda S, Hauffe T, Wilke T, Triebskorn R, et al. Intraspecific variation in cellular and biochemical heat response strategies of Mediterranean *Xeropicta derbentina [Pulmonata, Hygromiidae]*. PLOS ONE. 2014a;9: e86613.10.1371/journal.pone.0086613. 10.1371/journal.pone.0086613 PMC3903566 24475158

[27] Fischbach U, Köhler H-R, Wharam D, Gärtner U. Modeling the oxygen uptake, transport and consumption in an estivating terrestrial snail, *Xeropicta derbentina*, by the Colburn analogy. PLoS One. 2021;16(5):e0251379. 10.1371/journal.pone.0251379. 10.1371/journal.pone.0251379 PMC8136638 34014950

[28] Troschinski S, Dieterich A, Krais S, Triebskorn R, Koehler H-R. Antioxidant defense and stress protein induction following heat stress in the Mediterranean snail *Xeropicta derbentina* [Pulmonata, Hygromiidae]. J Educ Chang Exp Biol. 2014;2014(217):4399–405. 10.1242/jeb.113167 [Pulmonata, Hygromiidae. 10.1242/jeb.113167 25394630

[29] Dittbrenner N, Lazzara R, Köhler H-R, Mazzia C, Capowiez Y, Triebskorn R. Heat tolerance in Mediterranean land snails: histopathology after exposure to different temperature regimes. J Molluscan Stud. 2009;75(1):9–18. 10.1093/mollus/eyn033.

[30] Köhler H-R, Lazzara R, Dittbrenner N, Capowiez Y, Mazzia C, Triebskorn R. Snail phenotypic variation and stress proteins: do different heat response strategies contribute to Waddington’s widget in field populations? J Exp Zool Pt B. 2009;312B(2):136–47. 10.1002/jez.b.21253. 10.1002/jez.b.21253 19065565

[31] Scheil AE, Köhler H-R, Triebskorn R. Heat tolerance and recovery in Mediterranean land snails after pre-exposure in the field. J Molluscan Stud. 2011;77(2):165–74. 10.1093/mollus/eyr003.

[32] Triebskorn R, Mazzia C, Capowiez Y, Dittbrenner N, Zuern A, Koehler H-R. Cellular adaptation and recovery in response to heat stress in Mediterranean snails (*Xeropicta derbentina*, Theba pisana and cernuella virgata). Comp Biochem Physiol Mol Integr Physiol. 2008;151(1):S27–8. 10.1016/j.cbpa.2008.05.104.

[33] Seuront L, Ng TPT. Standing in the sun: infrared thermography reveals distinct thermal regulatory behaviours in two tropical high-shore littorinid snails. J Mollus Stud. 2016;82(2):336–40. 10.1093/mollus/eyv058.

[34] Seuront L, Ng TPT, Lathlean JA. A review of the thermal biology and ecology of molluscs, and of the use of infrared thermography in molluscan research. J Molluscan Stud. 2018;84(3):203–32. 10.1093/mollus/eyy023.

[35] Jeong SY, Tso CY, Wong YM, Chao CYH, Huang B. Daytime passive radiative cooling by ultra emissive bio-inspired polymeric surface. Sol Energy Mater Sol Cells. 2020;206:110296. 10.1016/j.solmat.2019.110296.

[36] Shi NN, Tsai C-C, Camino F, Bernard GD, Yu N, Wehner R. Keeping cool: enhanced optical reflection and radiative heat dissipation in Saharan silver ants. Science. 2015;349(6245):298–301. 10.1126/science.aab3564. 26089358 10.1126/science.aab3564

[37] Marion GM, Henry GHR, Freckman DW, Johnstone J, Jones G, Jones MH, et al. Open-top designs for manipulating field temperature in high-latitude ecosystems. Glob Change Biol. 1997;3(Suppl. S1):20–32. 10.1111/j.1365-2486.1997.gcb136.x.

[38] Welshofer KB, Zarnetske PL, Lany NK, Thompson LAE. Open-top chambers for temperature manipulation in taller-stature plant communities. Methods Ecol Evol. 2018;9(2):254–59. 10.1111/2041-210X.12863.

[39] Bar Z. Variation and natural selection in shell thickness of *Theba pisana* along climatic gradients in Israel. J Molluscan Stud. 1978;44:322–26.

[40] Hu C, Yang J, Qi Z, Wu H, Wang B, Zou F, et al. Heat shock proteins: biological functions, pathological roles, and therapeutic opportunities. MedComm. 2022;3(3):e161. 10.1002/mco2.161. 10.1002/mco2.161 PMC9345296 35928554

[41] Burton RF. Ca metabolism and acid-base balance in *helix pomatia*. In: Spencer Davies P, editor. Perspectives of experimental biology. Vol. 1. zoology, Oxford: Pergamon; 1976. p. 7–16.

[42] Heisler N. Acid-base regulation in animals. Amsterdam: Elsevier; 1986.

[43] De Meester L, Stoks R, Brans KI. Genetic adaptation as a biological buffer against climate change: potential and limitations. Integr Zool. 2018;13(4):372–91. 10.1111/1749-4877.12298. 29168625 10.1111/1749-4877.12298 PMC6221008

[44] Araújo MB, Ferri-Yáñez F, Bozinovic F, Marquet PA, Valladares F, Chown SL. Heat freezes niche evolution. Ecol Lett. 2013;16(9):1206–19. 10.1111/ele.12155. 23869696 10.1111/ele.12155

[45] Geerts AN, Vanoverbeke J, Vanschoenwinkel B, Van Doorslaer W, Feuchtmayr H, Atkinson D, et al. Rapid evolution of thermal tolerance in the water flea *Daphnia*. Nat Clim Change. 2015;5(7):665–68. 10.1038/nclimate2628.

[46] Franks SJ, Kane NC, O’Hara NB, Tittes S, Rest JS. Rapid genome-wide evolution in *Brassica rapa* populations following drought revealed by sequencing of ancestral and descendant gene pools. Mol Ecol. 2016;25(15):3622–31. 10.1111/mec.13615. 27072809 10.1111/mec.13615 PMC4963267

